# A robust SNP-haplotype assay for *Bct* gene region conferring resistance to *beet curly top virus* in common bean (*Phaseolus vulgaris* L.)

**DOI:** 10.3389/fpls.2023.1215950

**Published:** 2023-07-14

**Authors:** Alvaro Soler-Garzón, Deidrah Goldoff, Alyson Thornton, Kylie D. Swisher Grimm, John P. Hart, Qijian Song, Carl A. Strausbaugh, Phillip N. Miklas

**Affiliations:** ^1^ Irrigated Agriculture Research and Extension Center, Washington State University, Prosser, WA, United States; ^2^ Global Pathology Support Platform, HM Clause Seed Company, Davis, CA, United States; ^3^ Temperate Tree Fruit and Vegetable Research Unit, United States Department of Agriculture Agricultural Research Service (USDA-ARS), Prosser, WA, United States; ^4^ Tropical Agriculture Research Station, United States Department of Agriculture Agricultural Research Service (USDA-ARS), Mayagüez, Puerto Rico; ^5^ Soybean Genomics and Improvement Laboratory, United States Department of Agriculture Agricultural Research Service (USDA-ARS), Beltsville, MD, United States; ^6^ Northwest Irrigation and Soils Research Laboratory, United States Department of Agriculture Agricultural Research Service (USDA-ARS), Kimberly, ID, United States; ^7^ Grain Legume Genetics and Physiology Research Unit, United States Department of Agriculture Agricultural Research Service (USDA-ARS), Prosser, WA, United States

**Keywords:** geminivirus, dry bean, exonuclease V, genome-wide association study, marker-assisted selection, snap bean

## Abstract

Beet curly top virus (BCTV), which is synonymous with curly top virus (CTV), causes significant yield loss in common bean (snap and dry beans) cultivars and several other important crops. Common bean cultivars have been found to be resistant to CTV, but screening for resistance is challenging due to the cyclical nature of epidemics and spotty feeding by the leafhopper that vectors the virus. We used an SNP dataset for the Snap Bean Association Panel (SnAP) agro-inoculated with CTV-Logan (CA/Logan) strain to locate the *Bct* gene region to a 1.7-Mb interval on chromosome Pv07 using genome-wide association study (GWAS) analysis. Recombinant lines from the SnAP were used to further narrow the *Bct* region to a 58.0-kb interval. A missense SNP (S07_2970381) in candidate gene Phvul.007G036300 Exonuclease V (EXO5) was identified as the most likely causal mutation, and it was the most significant SNP detected by GWAS in a dry bean population (DBP) naturally infected by the CTV-Worland (Wor) strain. Tm-shift assay markers developed for SNP S07_2970381 and two linked SNPs, S07_2970276 and S07_2966197, were useful for tracking different origins of the *Bct* EXO5 candidate gene resistance to CTV in common bean. The three SNPs identified four haplotypes, with haplotype 3-1 (Haplo3-1) of Middle American origin associated with the highest levels of CTV resistance. This SNP-haplotype assay will enable breeders to track resistance sources and to develop cultivars with better CTV resistance.

## Introduction

Beet curly top virus (BCTV) can cause severe yield loss in susceptible cultivars of snap and dry beans (collectively known as common bean, *Phaseolus vulgaris* L.) and affects many other crops, including sugar beet (*Beta vulgaris* L.), pepper (*Capsicum annuum* L.), and tomato (*Solanum lycopersicum* L.) (Creamer, 2020). BCTV is endemic to the semi-arid regions of the western US, which are of paramount importance for producing high-quality and disease-free snap and dry bean seeds. BCTV is a single-stranded DNA virus of the genus *Curtovirus* within the family *Geminiviridae* and is transmitted in a persistent manner by the beet leafhopper (*Circulifer tenellus* B.). Among the 11 strains of BCTV described in the literature, Colorado (CO) and Worland (Wor) have been two of the more commonly isolated strains from plants and beet leafhoppers in recent studies in the western US. In contrast, the California/Logan (CA/Logan) strain has recently only been found in Idaho (Rondon et al., 2016; Strausbaugh et al., 2017; Giladi et al., 2020; Chiginsky et al., 2021; Hu et al., 2021; Swisher Grimm et al., 2021; Melgarejo et al., 2022; Rivedal et al., 2022) and has been distinct. The CA/Logan strain was the first to be collected and sequenced, while the CO and Wor strains have been described and established more recently (Stenger et al., 1990; Hormuzdi and Bisaro, 1993; Varsani et al., 2014; Strausbaugh et al., 2017). Endemics are cyclical in nature, with severity dependent on climatic events and their timing, which favor spring hatches of the beet leafhopper vector from weed host plants infected by BCTV, such as wild mustard (*Sinapis arvensis* L.), Russian thistle (*Salsola tragus* L.), and kochia (*Kochia scoparia* L. Schrad.) (Bennett, 1971).

BCTV [synonymous with curly top virus (CTV)] causes symptoms in common bean, including leaf curling, chlorosis, and severe plant stunting (Silbernagel and Morales, 2005). Early seedling infection of susceptible plants causes significant crop loss as seedlings typically wither and die. The reduced plant stands from early CTV infection mimic root rotting problems. Symptoms are generally less severe in susceptible bean plants infected at later growth stages and in plants with partial resistance, although seed yields may be significantly reduced. Seed treatments containing systemic insecticides provide good protection during the early seedling growth stages, but the most effective control is obtained by deploying natural genetic resistance in the host (Singh and Schwartz, 2010). The most effective and widely deployed resistance in common bean is conditioned by the major dominant *Bct* allele identified by Larsen and Miklas (2004) on chromosome Pv07 in a snap bean RIL population. Furthermore, the *Bct* locus, derived from the landrace ‘Jatu Rong’ (G122), was associated with a minor-effect quantitative trait locus (QTL) conferring resistance against CTV in the G122/’Taylor Horticulture’ dry bean RIL population (Larsen et al., 2010). The same study identified a major-effect QTL on Pv06 for CTV resistance. The *Bct* region was also associated with a major quantitative resistance effect against bean dwarf mosaic virus (BDMV) (Miklas et al., 2009).

Strategic implementation of host resistance in common bean has become a common practice for mitigating CTV risk to commercial seed production west of the US Continental Divide. Unfortunately, the selection for resistance, including that conditioned by the *Bct* allele, is difficult because field-based screening is unreliable due to the cyclical nature of epidemics. Additionally, there is a lack of uniform disease pressure during an epidemic because of spotty feeding by the leafhopper, as common bean is a non-preferred food source (Munyaneza and Upton, 2005), and BCTV strains can vary over time (Strausbaugh et al., 2017). Moreover, the virus is not mechanically transmissible, greatly complicating high throughput greenhouse testing. These constraints to screening for resistance to CTV clearly favor marker-assisted selection (MAS) as the most effective and efficient selection method.

Larsen and Miklas (2004) identified a sequence-characterized amplified region (SCAR) marker linked with *Bct* gene. This marker, known as ‘SAS8._1550_’, has led to greater accessibility and increased deployment of *Bct*. However, the SCAR ‘SAS8._1550_’, based on a polymorphism tightly linked to *Bct*, is subject to potential recombination events that can lead to false positives (selected plants carrying the marker allele for resistance but expressing a susceptibility phenotype) or false negatives (discarding plants that carry the marker allele for susceptibility but express a resistant phenotype). In addition, it was demonstrated that ‘SAS8._1550_’ had reduced diagnostic value in snap bean genotypes derived primarily from the Middle American gene pool (Larsen and Miklas, 2004), which includes a large complement of economically important germplasm.

Our research objectives aimed to i) acquire a comprehensive understanding of the genetic basis of the *Bct* allele and ii) enhance the efficiency and reliability of MAS for *Bct* in snap and dry bean germplasm.

## Materials and methods

### Plant materials

The Snap Bean Association Panel (SnAP) is comprised of 378 historic-to-modern cultivars that span both fresh market and processing types, including large sieve, small sieve, wax, bush blue lake, Kentucky, Romano, pole, and others. The SnAP includes public and private industry-released snap bean cultivars predominantly from the United States, some landrace cultivars, and a few breeding lines. The SnAP has recently been used for Genome-Wide Association Studies (GWAS) of herbicide tolerance (Saballos et al., 2022) and resistance to white mold (Arkwazee et al., 2022). The SnAP expands on the 149 snap bean accessions for the Snap Bean Diversity Panel (SBDP) that composed part of the BeanCAP (Common Bean Coordinated Agricultural Project) panel, which was comprised of both dry and snap beans (http://arsftfbean.uprm.edu/beancap/). The SBDP was used in a diversity analysis of snap bean (Wallace et al., 2018) and for GWAS of total phenolic content and root rot resistance (Myers et al., 2019; Huster et al., 2021). In addition to the SnAP, a dry bean population (DBP) comprised of 88 advanced inbred breeding lines and cultivars representing different market classes was included from the USDA-ARS bean breeding program in Prosser, WA.

### Phenotypic evaluations

Disease resistance or susceptibility reactions to CTV were obtained for 83 of the SnAP accessions from published information, including from the Agricultural Marketing Service-USDA scanned Plant Variety Protection (PVP) database, cultivar registration articles, and the Germplasm Resources Information Network (https://npgsweb.ars-grin.gov/gringlobal/search). An additional 289 SnAP accessions were phenotyped for CTV disease reaction using an infectious CA/Logan clone of CTV and an agro-inoculation screening procedure in a controlled growth room environment at Harris Moran Seed Company in Sun Prairie, Wisconsin. The CTV CA/Logan strain was cloned into the binary vector pBIN19 in *Agrobacterium tumefaciens* strain GV3111 (Ti plasmid pTiB6S3SE) obtained originally from Dr. Drake Stenger. This unpublished infectious clone had increased infectivity for agro-inoculation of common bean compared to earlier-developed clones (Stenger et al., 1990; Stenger et al., 1992).

For each of the 289 accessions, two pots with a diameter of 15 cm were filled with MetroMix-510 growing medium (Premier Horticulture, Inc., Red Hill, PA), and each pot contained two plants. The plants were covered and placed in a dark growth chamber for 24 h after inoculation. After 24 h, they were maintained in a growth chamber with a 14-/10-h light cycle and a temperature range of 18°C–27°C. The seedlings were transplanted into 1-gallon pots (two plants/pot) 2–6 days post-inoculation. The accessions were tested in subsets of approximately 30 accessions, in 4-day intervals, in a growth room. Each subset included two pots with two plants, each of the susceptible ‘Caprice’ and resistant ‘Hystyle’ cultivars as controls. Six accessions did not germinate, including ‘Angers’, ‘Processor’, ‘RH13’, ‘Serin’, ‘Golden Rod’, and ‘Sundial’. Disease reactions were recorded 17–24 days post-inoculation (dpi) as resistant (no visible symptoms), susceptible (with assorted symptoms: leaf rugosity, leaf cupping, leaf curling, plant stunting), or intermediate resistant (with fewer and less severe symptoms) (Larsen et al., 2010).

The DBP accessions were sown during the summer of 2022 in two replications at the Washington State University (WSU) Research Station—Roza Unit (46°17′43.6ʺN 119°43′48.0ʺW) in Prosser, WA, USA, where CTV is endemic. The experimental design was a randomized complete block, and each plot consisted of four rows of 3-m row length, with a spacing of 0.56 m between rows. To determine the CTV strain(s) causing infection in the trial, leaf tissue was collected from 1 asymptomatic plant and 16 symptomatic plants during the early pod-fill R3 growth stage. The tissue was extracted using a modified Dellaporta protocol (Crosslin et al., 2006) and analyzed by conventional PCR using the strain-specific primers previously described in Strausbaugh et al. (2008) to detect three strains of CTV, namely, the CFH strain [also referred to as beet severe curly top virus (BSCTV)], Wor strain [beet mild curly top virus (BMCTV)], and CA/Logan strain. All PCR reactions consisted of 5 µl of 5× GoTaq Green PCR buffer (Promega Corp, USA), 0.25 µl 10 mM dNTP (each), 0.5 µl of 20 µM forward and reverse primers, 17.65 µl H_2_O, 1 µl of total nucleic acid extract, and 0.1 µl GoTaq DNA polymerase (Promega Corp., USA). Thermal cycler conditions consisted of 95°C for 2 min, followed by 40 cycles of 95°C for 1 min, 54°C for 1 min, and 72°C for 1 min, and a final extension step of 72°C for 5 min. Amplicons were visualized on a 1.5% agarose gel with ethidium bromide staining.

Disease reaction was rated 60 days after planting on a scale from 1 to 9, where 1 = no symptoms and 0% of plants infected, 5 = moderate disease symptoms and up to 30% of plants infected, and 9 = severe disease symptoms with ≥80% of the plants dead or dying (Larsen and Miklas, 2004). Trait data were spatially corrected, and best linear unbiased predictors (BLUPs) were calculated using a two-dimensional P-spline mixed model with the Mr.Bean web application (Aparicio Arce, 2018) and the R package SpATS v1.0-11 (Rodríguez-Álvarez et al., 2018).

### Genotyping

DNA was extracted from each SnAP accession using the Qiagen Plant DNeasy Mini Kit (Hilden, Germany) and diluted to 10 ng/μl with nuclease-free water. Multiplexed genotyping-by-sequencing (GBS) libraries were generated through digestion with ApeKI following the protocol and with barcodes detailed in Elshire et al. (2011). Single-end sequencing of multiplex GBS libraries was performed using the Illumina HiSeq2000 (Illumina, San Diego, CA, USA). The barcodes of raw Illumina sequence were trimmed, and reads were aligned to the reference Andean G19833 Phaseolus vulgaris v2.1 genome sequence (https://phytozome-next.jgi.doe.gov/info/Pvulgaris_v2_1) using Bowtie2 v2.4.1 (Langmead and Salzberg, 2012). The alignments in BAM format were sorted by SAMtools v1.9 (Li et al., 2009).

SNP discovery and genotype calling were conducted using NGSEP v3.3.0 (Next Generation Sequencing Experience Platform) pipeline (Perea et al., 2016) with G19833 *Phaseolus vulgaris* v2.1 as reference genome (Schmutz et al., 2014). Maximum base quality score was set to 30, and the quality minimum was set to 40 for reporting a variant. All SNP markers detected with <40% missing values, and a minor allele frequency (MAF) of 0.01 were retained to perform imputation with the *ImputeVCF* module in NGSEP, which is a reimplementation of the Hidden Markov Model (HMM) implemented in the package fastPHASE (Scheet et al., 2006). Annotation of variants was performed using the command *Annotate* by NGSEP.

For the DBP accessions, the leaf tissue was collected from individual plants grown in the USDA-ARS greenhouses at Prosser, WA. Genomic DNA was isolated from 20 mg of the leaf tissue using a Qiagen DNeasy 96 Plant Kit (Hilden, Germany) and sent to the US Department of Agriculture Agricultural Research Service (USDA-ARS), Soybean Genetics and Improvement Laboratory, Beltsville, MD, for the 11,292 SNPs BARC Illumina chip assay (Miklas et al., 2020). The SNP genotyping was conducted on the Illumina platform following the Infinium HD Assay Ultra protocol (Illumina Inc., San Diego, CA) (Song et al., 2015). The SNP positions were updated by alignment to the v2.1 reference genome assembly of G19833 and were filtered based on a minimum allele frequency (MAF) of 0.01 and a missing data rate of <20%.

### Population genetic analysis

The SNP data sets used for population structure analysis in SnAP and DBP were adjusted by linkage disequilibrium (LD) pruning using a 0.5 r^2^ threshold and the *–indep-pairwise* function in Plink v2.0 (https://www.cog-genomics.org/plink/2.0/), with a sliding window of 50 kb shifting by five bases. In addition, two dry bean lines, ‘BAT 93’ (representing the Middle American gene pool) and ‘Jalo EPP 558’ (representing the Andean gene pool), were added to assess the structure and diversity in SnAP. The sequence data for ‘BAT 93’ and ‘Jalo EPP 558’ was obtained by GBS, as described above.

The pruned set of SNPs with <20% missing values and a MAF > 0.01 were retained. Population structure was estimated using a Bayesian Markov chain Monte Carlo model (MCMC) implemented in STRUCTURE 2.3 software (Pritchard et al., 2000). Ten runs (k) were performed for each number of populations set from 1 to 10. Burn-in time and MCMC replication number were set to 100,000 and 200,000, respectively. The most likely number of populations (K) in the SnAP was calculated with the Evanno method (Evanno et al., 2005) using Pophelper v2.3.1 (Francis, 2017). Finally, the merge Q-matrix was obtained using the CLUMPP v1.1 software (Jakobsson and Rosenberg, 2007) to determine the robustness of the population assignments at each K.

### GWAS

GWAS analysis was conducted by mixed linear model (MLM) using the genome association and prediction integrated tool (GAPIT) (Lipka et al., 2012). An identity-by-state kinship matrix was created using the Efficient Mixed Model Association (EMMA) algorithm implemented in GAPIT R package with corrections for kinship and population structure. Five principal components (PCs) generated from GAPIT were included as covariates, and the Bonferroni procedure was applied to control the experiment-wise type I error rate at 0.05. The GWAS results were visualized using CMPlot v3.62 (Yin, 2016) and IntAssoPlot v3 (He et al., 2020) for a graphical representation of regional-based marker–trait associations.

### Candidate gene identification

Candidate genes annotated within the *Bct* region were identified using the gene models from G19833 v2.1 common bean reference genome. Selected candidate genes were amplified and sequenced in a subset of 14 snap and 14 dry bean genotypes from Andean and Middle American gene pools.

A set of primers were designed to obtain full-length candidate genes using Primer3 software (Koressaar and Remm, 2007) (http://bioinfo.ut.ee/primer3-0.4.0/). The PCR volume was 25 µl, containing 1× Taq buffer, 1.8 mM of MgCl_2_, 0.4 mM of dNTPs mix (Promega^®^, Madison, WI), 0.25 µM of each primer (forward and reverse), 1 U of Taq polymerase (Promega^®^, Madison, WI), and 25 ng of genomic DNA. The amplification conditions were 2 min at 95°C, followed by 38 cycles at 94°C for 20 s, 57°C for 30 s, and 72°C for 90 s, and a final extension at 72°C for 5 min, performed in a PCR Eppendorf Mastercycler (Eppendorf AG, Hamburg, Germany). PCR products were visualized by gel electrophoresis on 2% (w/v) agarose. Sanger sequencing from PCR products obtained was performed using the services of Eurofins MWG Operon (Louisville, KY, USA). Sequencing data were analyzed using Geneious 9.1.8 (Kearse et al., 2012), which was used for sequence trimming, alignment, and variant detection.

### Development of single nucleotide polymorphism markers in the *Bct* region

A set of five SNPs and one InDel marker were developed from a variants catalog obtained from Lobaton et al. (2018) and used to refine the *Bct* interval identified by GWAS in SnAP (**Supplementary Table S1**). From the refined interval, three nonsense variants (S07_2966197, S07_ 2970276, and S07_ 2970381) were selected in candidate gene sequences based on polymorphism among 14 snap and 14 dry beans genotypes and converted to markers using the allele-specific method (Wang et al., 2005). These three variants (S07_2966197, S07_ 2970276, and S07_ 2970381), and the original ‘SAS8._1550_’ SCAR marker, using the protocol described by Larsen and Miklas (2004), were assayed in the SnAP and DBP. Lastly, three additional SNP markers (Pv07_3044767, Pv07_331344, and Pv07_3644749), identified by Ariza-Suarez et al. (2023) to be linked with resistance to bean leaf crumple virus (BLCrV) in proximity to the *Bct* region, were converted to Tm-shift assays for genotyping the DBP (**Table S1**). BLCrV is a single-stranded DNA Begomovirus in family Geminiviridae.

Tm-shift SNP markers were amplified by PCR on an Eppendorf Mastercycler (Eppendorf AG, Hamburg, Germany). The PCR volume was 20 μl containing 20 ng of genomic DNA, 1× Taq buffer, 1.5 mM of MgCl_2_, 0.2 mM of dNTPs mix (Promega), 0.15 μM of each primer (two allele-specific forward primers and the common reverse primer), 1× EvaGreen^®^ dye, and 0.1 μl of Taq polymerase (Promega) under the following thermal profile: 94°C for 2 min, then 38 cycles of denaturation at 92°C for 20 s, annealing for 20 s (the temperature was specific to each primer trio), and extension at 72°C for 20 s, and final extension at 72°C for 5 min. Melting point analysis for allele determination of the PCR products was performed with a fluorescence-detecting thermocycler (LightCycler^®^ 4890 Instrument II, Roche, Basal Switzerland). EvaGreen Fluorescent detection was performed for 1 min at 95°C, and the melting curve step ramped from 65°C to 95°C in increments of 1°C every 20 s.

### Identification of haplotypes in *Bct* candidate gene in DBP accessions

The S07_2966197, S07_ 2970276, and S07_ 2970381 markers were used to identify haplotypes correlated with quantitative field reaction to CTV in the DBP. An analysis of variance between haplotypes was carried out by F_trimmed-means_ one-way ANOVA using the ggbetweenstats function in the R-Package ggstatsplot. Yuen’s trimmed means test was implemented using Robust *p-*value fitting method using the ggbetweenstats function in the R-Package ggstatsplot (Patil, 2021). Overall, the F_trimmed-means_ method provides a robust approach for analyzing data that may contain outliers and can help identify statistically significant differences between groups.

## Results

### Curly top virus screening

Out of the 289 SnAP accessions screened by agro-inoculation with the CTV CA/Logan strain, 166 accessions were resistant, 66 were susceptible, 4 showed intermediate resistance, and 53 were segregating with plants exhibiting either resistant or susceptible (20 accessions); resistant or intermediate resistance (18 accessions); or resistant, intermediate resistant, or susceptible responses (15 accessions). The disease reactions for 83 SnAP accessions, compiled from various public information sources, were categorized as 42 resistant, 32 susceptible, and 9 intermediate resistant to CTV. For upstream analysis, the qualitative reactions of each accession were transformed into a quantitative score, with resistant being coded as 1, susceptible as 0, and intermediate resistant as 2 (**Supplementary Table S2**).

In addition, 88 DBP accessions were evaluated for CTV reaction (scored from 1 to 9) under natural field infection. Based on the BLUP values for CTV reaction, 43 accessions were resistant (disease score between 1 to 4), 33 were tolerant (between 4.1 to 6.9), and 14 were susceptible (between 7 to 9) to the Wor strain 60 days after planting (**Supplementary Table S3**). PCR analysis with strain-specific primers detected the Wor strain in leaf tissue collected from this naturally infected field. Specifically, one asymptomatic plant and 11 of 16 symptomatic plants amplified a product of the expected size (397 bp) with the Wor strain primers.

### Population structure

According to STRUCTURE analysis of the SnAP (378 accessions), using a pruned set of 1,833 SNPs, a likelihood of K=2 was estimated, indicating the presence of two sub-populations belonging to the Andean and Middle American gene pools, as confirmed by gene-pool checks ‘Jalo EEP 558’ and ‘BAT 93’ (**Supplementary Figure S1**). Accessions were further categorized based on a membership coefficient of Q ≥ 0.85. The Andean population comprised 166 accessions (43.9%), and the Middle American, 27 accessions (7.1%). The remaining accessions were grouped as admixtures according to gene-pool predominancy, using a membership coefficient Q ≥ 0.5, with 108 accessions as Andean admixtures (28.6%) and 77 as Middle American admixtures (20.4%) (**Supplementary Table S4A**).

A similar STRUCTURE analysis was performed in the DBP (88 accessions), using an LD-pruned set of 974 SNP markers, and K=2 was estimated (**Supplementary Figure S2**), with 59 Andean (67.0%) and 29 Middle American (33.0%) accessions (**Supplementary Table S4B**). Gene pool classification was based on previous diversity studies (Cichy et al., 2015; Moghaddam et al., 2016).

### GWAS in SnAP and DBP

A filtered set of 25,472 biallelic SNPs from 378 SnAP accessions were obtained by GBS. Multiple GWAS analyses were conducted to identify the genomic regions associated with resistance to CTV in the SnAP. The first GWAS compared 208 resistant, 13 intermediate-resistant, and 98 susceptible SnAP accessions, and 53 accessions segregating for CTV symptoms were excluded. A significant interval from 2.79 to 4.32 Mb on chromosome Pv07 was identified, exceeding the Bonferroni-corrected significance threshold of α = 0.05 (*p* = 1.7E−36), which was associated with resistance and intermediate resistance to CTV (**Supplementary Figure S3A**). This interval corresponds with the location of the *Bct* gene that was identified by Larsen and Miklas (2004) in a snap bean RIL population and a QTL (BCT7.1^GT^) that conditions resistance to CTV found in the dry bean landrace G122 named ‘Jatu Rong’ (PI 163120) from India (Larsen et al., 2010).

**Figure 1:**
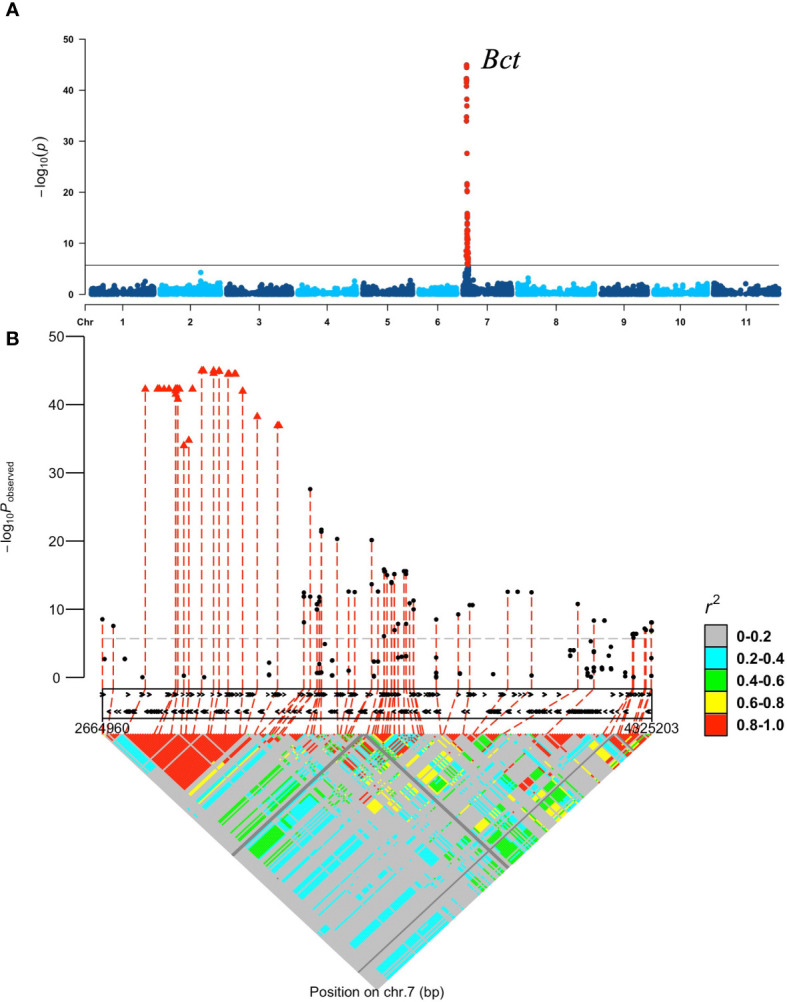
GWAS for CTV reaction by agro-filtration (Logan strain) in the SnAP, accessions with resistant and intermediate resistant phenotypes coded 1 versus susceptible lines coded 0: **(A)** Manhattan plot produced by GWAS analysis with GAPIT. Each dot represents each SNP obtained by GBS after filtering. The horizontal line represents a significance level at p-value < 1.96E−06. The peak region corresponds with the previous location for the *Bct* gene and BCT7.1 QTL (Larsen and Miklas, 2004; Larsen et al., 2010); **(B)** 128 significant SNPs from GWAS, which define the *Bct* gene region on Pv07. The x-axis depicts candidate genes (black arrows) within the 1.6-Mb region. Pairwise linkage disequilibrium using the significant SNPs for the CTV resistance region identifies one LD block with 50 significant SNPs (red triangles) with the highest p-value ranging from 1.08E−34 to 1.12E−45 [−log10(p-value) = 33.97 to 44.95] in an interval from 2,794,761 to 3,199,136 bases on Pv07.

A separate GWAS comparing 208 resistant with 98 susceptible accessions and 13 intermediate-resistant with 98 susceptible accessions revealed similar significant peak intervals of 2.66–4.32 Mb (*p* = 7.81E−44) and 2.79–3.60 Mb (*p* = 1.89.17) on Pv07, respectively (**Supplementary Figures S3B, C**). No significant SNPs were detected by the GWAS comparing 208 resistant and 13 intermediate-resistant SnAP accessions. Therefore, a GWAS was conducted comparing 239 resistant and intermediate-resistant accessions coded as 1 with 98 susceptible accessions coded as 0. This analysis showed a genomic interval (containing 129 significant SNPs) from 2.66 to 4.32 Mb on chromosome Pv07 with significantly increased significance (*p* =1.12E−45), as expected for a single major allele (**Figure 1A**). One LD block within the 2.66–4.32 interval, from 2,794,761 to 3,199,136 bases (**Supplementary Table S5**), consisting of 50 significant SNPs with the highest *p*-values ranging from *p* = 1.08E−34 to 1.12E−45, became the target region for the *Bct* allele for CTV resistance on Pv07 (**Figure 1B**). Other LD blocks with CTV resistance associations less than *p* = 2.43E−28 were excluded as targets within the 2.66 – 4.32 Mb interval.

**Figure 2:**
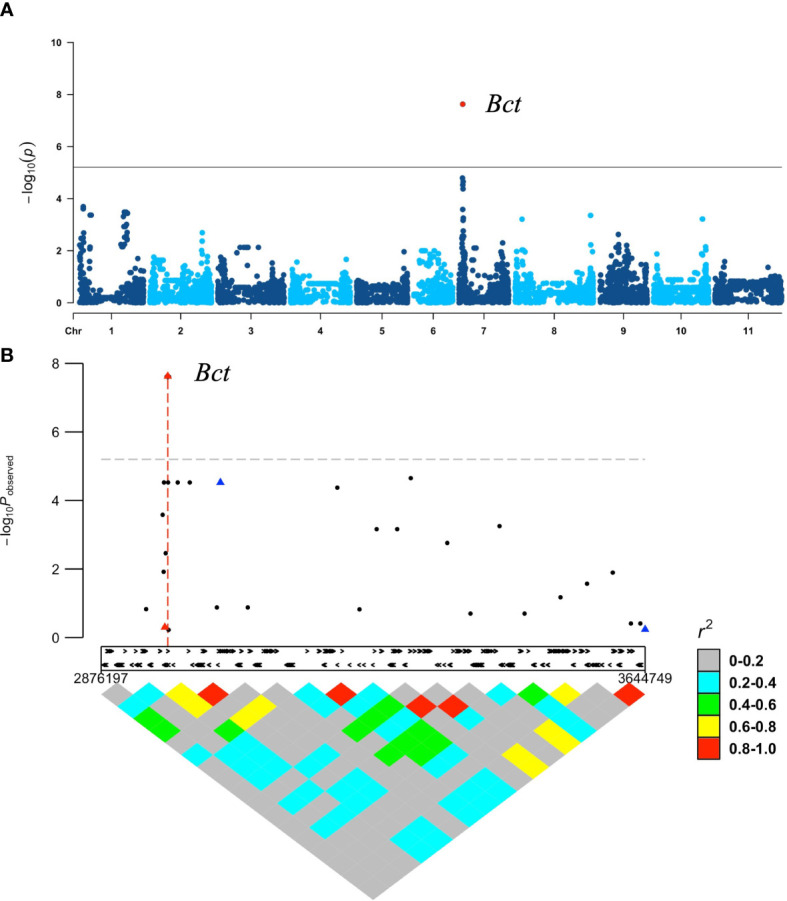
GWAS in DBP evaluated for CTV reaction (Worland strain) under field conditions using 7,983 SNP markers obtained from 12_K BARC Illumina chip assay with six additional select Tm-shift assay SNP markers: **(A)** Manhattan plot produced by GWAS analysis with GAPIT. Only one significant SNP S07_2970381 (red dot) for *Bct* region was identified; **(B)** low LD, and no associations were observed between *Bct* region (red triangle) and SNP markers (blue triangles) linked to BLCrV resistance.

GWAS with the 88 DBP accessions, using 7,983 SNP markers after filtering, did not detect any significant peaks for resistance to CTV. To improve the analysis, we incorporated six additional SNP markers, including three identified from sequencing candidate genes in the *Bct* region described below and three associated with resistance to bean leaf crumple virus (BLCrV) from Ariza-Suarez et al. (2023). Using these additional SNPs, the GWAS analysis identified a missense SNP, S07_2970381 (G19833v2.1 reference genome), within a candidate gene in *Bct* region with a high *p-*value (*p* =1.84E−07) (**Figure 2A**). The three SNP markers linked to BLCrV resistance (**Figure 2B**) had low LD with the s*Bct* region and did not associate with CTV resistance.

### Candidate gene identification in *Bct* region

**Figure 3:**
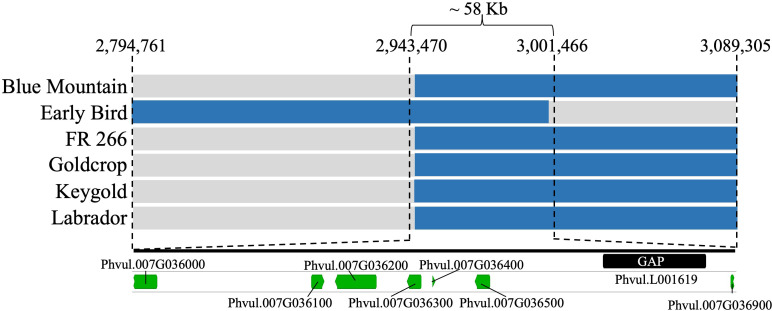
SnAP accessions with recombinant haplotype in *Bct* region, located seven gene models (green bars) distributed in 58 kb, and one additional gene model in Scaffold 1137 located in a gap (black bar) region in G19833 v2.1 reference genome. Due to presence of the susceptible allele from the S07_2943470 and S07_3001466 SNP markers in the six recombinant lines, the Phvul.007G036000 and Phvul.007G036900 gene models were excluded. More details can be found in **Supplementary Table S6**.

Six resistant SnAP accessions displayed recombination within the targeted LD block (2,794,761–3,199,136 bp) for the *Bct* region on Pv07 (**Supplementary Table S6**). These accessions were ‘Goldcrop’ (SnAP152) and ‘Keygold’ (SnAP191) from the Andean gene pool; ‘Blue Mountain’ (SnAP037) from the Middle American gene pool; and Andean admixtures ‘Early Bird’ (SnAP107), ‘FR 266’ (SnAP136), and ‘Labrador’ (SnAP194). Haplotypes for the six developed markers spanning the LD block in the recombinant SnAP accessions narrowed the interval for the *Bct* region to 58.0 kb from 2,943,470 to 3,001,466 bases (**Figure 3**; **Supplementary Table S6**).

Six candidate genes were annotated in the refined 58.0 kb *Bct* region based on the Andean G19833 common bean v2.1 gene models (**Table 1**). However, one of these gene models, Phvul.L001619, was missing from a gap segment (Pv07: 2,988,789–2,998,791 bases) in the *Bct* region of G19833 v2.1. A BLASTn search in the previous version of the G19833 v1.0 reference genome revealed that this gene model had 100% similarity with the former Phvul.007G036700 in G19833 v1.0.

**Table 1:** Candidate gene models in G19833 v2.1 reference genome for the narrowed 58-kb interval for *Bct* gene region in the SnAP.

Gene Models G19833V2.1	Chr. G19833V2.1	Start (bases)	End (bases)	Sense	*Arabidopsis thaliana* homolog (*E*-value/%Identity)	Predicted function
Phvul.007G036100	Pv07	2,960,615	2,961,929	+	No hits found	Fasciclin-Like Arabinogalactan Protein 20-Related
Phvul.007G036200	Pv07	2,962,903	2,966,983	−	AT1G71110 (0.0/64%)	Transmembrane Protein
Phvul.007G036300	Pv07	2,969,877	2,971,300	−	AT5G60370 (1.76E−82/43%)	Exonuclease V (EXO5)
Phvul.007G036400	Pv07	2,972,341	2,972,661	+	AT1G49700 (1.68E−5/43%)	Unknown function
Phvul.007G036500	Pv07	2,976,416	2,977,924	−	AT2G01290 (1.16E−134/71%)	Ribose-5-Phosphate Isomerase 1-Related
Phvul.L001619 - Phvul.007G036700*	Scaffold 1137	407	844	−	AT2G01300 (3.78E−43/52%)	Mediator of RNA Polymerase II Transcription Subunit

BLASTP was performed in Arabidopsis thaliana using Arabidopsis Information Resource (TAIR) database.

*Gene model identified previously in G19833 v1.0.

Interestingly, two candidate genes in the *Bct* region, Phvul.007G036300 encoding a 5′ deoxyribonuclease domain-containing protein (Exonuclease V) and Phvul.L001619, which functions as a mediator of RNA polymerase II transcription subunit, showed homology with gene models from other plant species involved in the replication of geminiviruses (Hanley-Bowdoin et al., 2013). Another candidate gene, Phvul.007G036200 (transmembrane protein), may play a role in defense response to other organisms based on its homology to the *Arabidopsis thaliana* gene AT1G71110. Target open reading frame (ORF) sequencing of the three candidate genes revealed 29 variants, 28 SNPs, and 1 InDel among a subset of 28 snap and dry bean genotypes from diverse backgrounds (**Supplementary Table S7**). Additionally, Phvul.007G036400 and Phvul.007G036500 gene models within the *Bct* region were sequenced, with two nonsense InDel variants and two synonymous SNP variants identified, respectively. Overall, for the 33 variants in the five candidate genes assayed across the 28 genotypes, only one variant, a missense SNP on Pv07: 2,970,381 bases in candidate gene Phvul.007G036300, was completely associated with BCTV resistance.

The *in silico* analysis of the Exonuclease V (EXO5) protein of the sequenced ORF for gene Phvul.007G036300 did not uncover any disruptions in the EXO5 domain. Nevertheless, the missense SNP variant located in Pv07: 2,970,381 bases involves a transition of Adenine (A) to Thymine (T) base, resulting in radical replacement of a polar, uncharged amino acid, glutamine (Q), with a non-polar, hydrophobic amino acid, leucine (L), at position 279 in the EXO5 domain (i.e., p.Q279L) (**Table 2**).

**Table 2:** Polymorphisms detected within the ORF for *Bct* candidate gene Phvul.07G36300 among 28 genotypes—6 susceptible (S), 16 resistant (R), 2 intermediate resistance (IR), and 1 tolerant (T) to CTV conditioned by *Bct*.

Genotype	Gene pools	CTV reaction	Pv07: 2,970,037	Pv07: 2,970,125	Pv07: 2,970,276	Pv07: 2,970,288	Pv07: 2,970,312	Pv07: 2,970,329	Pv07: 2,970,381	Pv07: 2,970,452	Pv07: 2,970,576	Pv07: 2,970,668	Pv07: 2,970,835	Pv07: 2,970,858
			^a^Missense	synonymous	Missense	Missense	Missense	synonymous	Missense	synonymous	Missense	synonymous	Missense	Missense
			^b^F/L	A	S/C	R/M	R/M	I	Q/L	V	R/K	L	G/R	C/Y
G19833	Andean	NT	A	T	G	C	C	G	T	C	C	C	C	C
Gina	Andean	S	A	T	G	C	C	G	T	C	C	C	C	C
Navarro	Andean	S	A	T	G	C	C	G	T	C	C	C	C	C
Rocdor	Andean	S	A	T	G	C	C	G	T	C	C	C	C	C
Titan	Andean	S	A	T	G	C	C	G	T	C	C	C	C	C
Top Crop	Andean	S	A	T	G	C	C	G	T	C	C	C	C	C
Trend	Andean	S	A	T	G	C	C	G	T	C	C	C	C	C
Shannon	Andean	R	A	T	G	C	C	G	A	C	C	C	C	C
G122	Andean	R	A	T	G	C	C	G	A	C	C	C	C	C
Amity	Andean	R	A	T	G	C	C	G	A	C	C	C	C	C
Fortex	^c^MA admixed	R	A	T	G	C	C	G	A	C	C	C	C	C
Astun	Andean	IR	G	A	C	A	A	A	A	A	T	T	T	T
Early Bird	Andean	R	G	A	C	A	A	A	A	A	T	T	T	T
Cardinal	Andean	R	G	A	C	A	A	A	A	A	T	T	T	T
Profit	Andean	R	G	A	C	A	A	A	A	A	T	T	T	T
Tenderlake	Andean	R	G	A	C	A	A	A	A	A	T	T	T	T
Dubbele Witte	MA	IR	G	A	C	A	A	A	A	A	T	T	T	T
XAN176	MA	NT	G	A	C	A	A	A	A	A	T	T	T	T
DOR364	MA	NT	G	A	C	A	A	A	A	A	T	T	T	T
BAT 93	MA	R	G	A	C	A	A	A	A	A	T	T	T	T
Matterhorn	MA	R	G	A	C	A	A	A	A	A	T	T	T	T
UI 59	MA	R	G	A	C	A	A	A	A	A	T	T	T	T
Common Red Mexican	MA	R	G	A	C	A	A	A	A	A	T	T	T	T
Othello	MA	R	G	A	C	A	A	A	A	A	T	T	T	T
UI 111	MA	R	G	A	C	A	A	A	A	A	T	T	T	T
UI 114	MA	R	G	A	C	A	A	A	A	A	T	T	T	T
UI 123	MA	R	G	A	C	A	A	A	A	A	T	T	T	T
Sanilac	MA	T	G	A	C	A	A	A	A	A	T	T	T	T

Note that three genotypes were not tested (NT). The resistant allele of SNP S07_2970381 (G19833 v2.1 reference genome) co-segregates with the CTV phenotype and is highlighted in green.

a Variant type.

b Amino-acid substitutions in protein-coding regions.

c Middle American gene pool.

**Figure 4:**
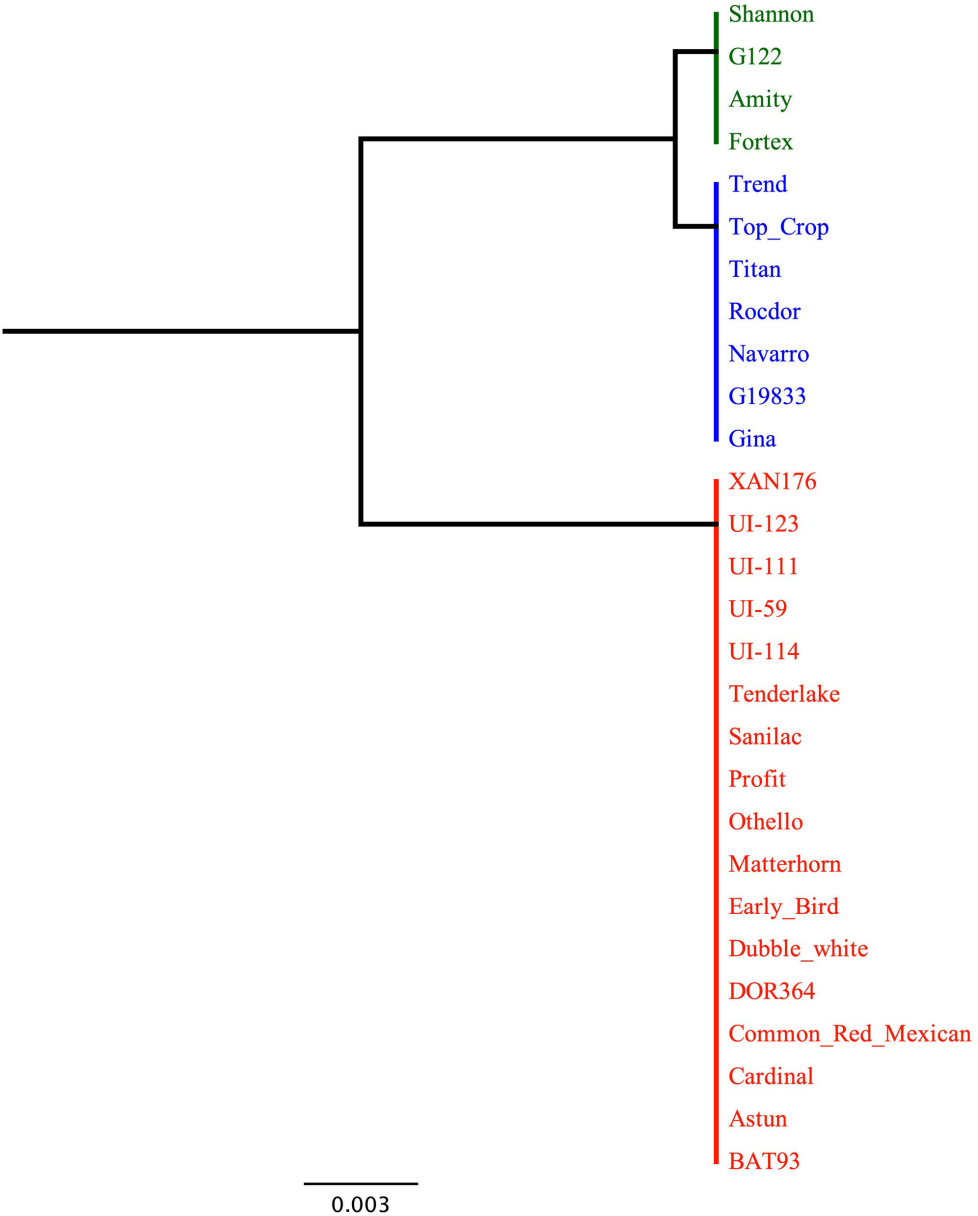
Dendrogram based on genetic distance for sequence diversity for candidate gene Phvul.007G036300 among 28 select SnAP and dry bean genotypes; 19 with and 6 without CTV resistance conditioned by *Bct*. Green cluster represents Andean susceptible genotypes, blue cluster Andean resistant (except for Middle American ‘Dubbele Witte’) and red cluster Middle American resistant to CTV. Blosum62 for alignment. Genetic distance Jukes-cantor. Tree build method UPGMA. Note three genotypes untested for CTV reaction were included: reference genome ‘G19833’ and ‘DOR 364’ and ‘XAN 176’ parents of the DX RIL population used by Soler-Garzón et al. (2021) to map BGY7.1 QTL for resistance to BGYMV.

The dendrogram based on the genetic distance of the translated protein for the *Bct* candidate gene Phvul.007G036300 in the 28 sequenced genotypes showed one cluster for susceptibility and two distinct clusters for resistance/intermediate resistance to CTV (**Figure 4**). The first cluster consisted of susceptible snap bean genotypes from the Andean gene pool. The second cluster included the resistant Andean snap bean genotypes ‘Shannon’, ‘Amity’, ‘Fortex’, and the Andean dry bean ‘G122’. The third cluster included the resistant Andean snap bean genotypes ‘Early Bird’, ‘Astun’, ‘Profit’, ‘Tenderlake’, the Middle American snap bean ‘Dubbele Witte’, and all the sequenced dry beans from the Middle American gene pool. The *Bct* EXO5 candidate gene protein sequence exhibits 99.75% identity between Andean-resistant and Andean-susceptible genotypes, with only one amino acid change observed for the codon containing SNP S07_2970381. In contrast, resistant and tolerant Middle American genotypes display 100% identity for the EXO5 protein.

### Tm-shift assays for diagnostic SNPs in *Bct* region

A Tm-shift assay named S07_2970381 was specifically designed to detect the missense SNP Pv07: 2,970,381 in the *Bct* candidate gene Phvul.007G036300 (EXO5), which exhibited polymorphism between resistant and susceptible genotypes to CTV. Additionally, two Tm-shift assays were developed to distinguish introgression haplotypes of the *Bct* region from Andean and Middle American gene pools. The first assay, named S07_2970276, targeted the missense SNP variant Pv07: 2,970,276 bases within Phvul.007G036300 gene and differentiated genotypes from Andean and Middle American gene pools. The second assay, named S07_2966197, focuses on another missense SNP variant, Pv07: 2,966,197 bases, found in the Phvul.007G36200 gene model by Soler-Garzón et al. (2021) in a race Durango dry bean diversity panel. This variant differentiated genotypes within the Middle American genepool. The primer sequences and Tm-shift melting curves for each SNP marker can be found in **Table 3** and **Supplementry Figure S4**, respectively.

**Table 3:** Primers used to assay select SNP markers for haplotyping the *Bct* EXO5 candidate gene region.

Tm shift assay markers	Alleles	Position (bases)^a^	Gene Model G19833 v2.1	Annotation	SNP type	Strand	Primer sequences	Sense 5′–3′	Ta
**S07_2966197**	A/G	Pv07: 2,966,197	Phvul.007G036200	Transmembrane Protein	Missense	Fa	gcgggcGGGCCTAAAGGTGAGGATGT	−	68
						R	CTCGCAGAATGTCAGGCCTATT		
						Fb	gcgggcagggcggcGGCCTAAAGGTGAGGATGC		
**S07_2970276**	G/C	Pv07: 2,970,276	Phvul.007G036300	Exonuclease V (EXO5)	Missense	Fa	gcgggcagggcggcATATCGCAACACTAACATATCATTAG	+	58
						R	CATCATGATTTCCCATGCAA		
						Fb	gcgggcATATCGCAACACTAACATATCATTAC		
**S07_2970381**	T/A	Pv07: 2,970,381	Phvul.007G036300	Exonuclease V (EXO5)	Missense	Fa	gcgggcagggcggcTGAATCCTCGTGCCACTCA	−	58
						R	GCTGTGAATCCAGACTCGTAGCA		
						Fb	gcgggcTTGAATCCTCGTGCCACTCT		

a Reference genome G19833 v2.1.

### *Bct*-linked markers assays

The S07_2970381 marker exhibited a 100% correlation with the CTV phenotype observed in SnAP. For the 378 SnAP accessions, 238 (63.0%) showed resistance or intermediate resistance to the CTV CA/Logan strain and possessed the resistance allele of the S07_2970381 marker, and 100 (26.5%) were susceptible and possessed the susceptible allele of the S07_2970381 marker, while 33 (8.7%) were segregating for CTV reaction, and 7 (1.8%) had missing data (**Supplementary Table S8**).

For the DBP (88 accessions) infected with the Wor strain, a correlation of 99% was observed between the S07_2970381 SNP marker and the disease score. There were 42 accessions with resistant disease scores ≤ 4.0, which possessed the resistant allele of the S07_2970381 SNP marker, and 13 with susceptible disease scores from 7.0 to 9.0, which possessed the susceptible allele (**Supplementary Table S9**). The 31 tolerant accessions, with disease scores from 4.1 to 6.9, possessed the resistant marker allele with two exceptions: ‘SVS-0683’ (DBP42) with a disease score of 6.3 and ‘Beluga’ (DBP45) with a disease score of 6.2, possessed the susceptible marker allele. Note that the ‘Beluga’ genotype exhibited a susceptible reaction when agro-infiltrated with Logan strain, while SVS-0863 was untested.

A comparison of genotypes for the S07_2970381 SNP marker and the ‘SAS8_.1500_’ SCAR marker developed by Larsen and Miklas (2004) showed a 97.1% similarity in the SnAP and 47.4% in the DBP. The ‘SAS8_.1550_’ SCAR marker, Pv07: 2,975,432–2,977,000 bases, is 4.1 kb downstream of the EXO5 candidate gene Phvul.007G036300 and in high linkage disequilibrium (LD) with the S07_2970381 and S07_2970276 SNP markers in the SnAP and DBP. In fact, ‘SAS8_.1550_’ and S07_2970276 markers were 100% identical in both bean panels.

### *Bct* region haplotypes

The SnAP and DBP were assayed for the S07_2970381, S07_2970276, and S07_2966197 markers to identify haplotypes for the *Bct* region. Four haplotypes were identified: Haplo1 with AGT sequence for the S07_2966197 (A/G), S07_2970276 (G/C), and S07_2970381 (T/A) SNPs, respectively; Haplo2 with AGA sequence; and Haplo3 divided into two sub-haplotypes, Haplo3-1 with ACA sequence and Haplo3-2 with GCA sequence, based on polymorphism for S07_2966197 in Middle American accessions.

In the SnAP (**Table 4**), Haplo1 was found in all 98 susceptible accessions: 97% Andean and Andean admixtures and 3% Middle American and Middle American admixtures. Haplo2 was a rare haplotype found in only five resistant accessions, four Andean/Andean admixtures, and one Middle American admixture. Haplo3-1 was observed in 162 resistant/intermediate resistant accessions: 76% Andean and Andean admixtures and 24% Middle American and Middle American admixtures. Haplo3-2 was found in 71 resistant/intermediate resistance accessions: 68%) Middle American and Middle American admixtures and 32% Andean and Andean admixtures.

**Table 4:** Haplotypes identified in SnAP (336 Accessions) phenotyped by agro-inoculation with Logan strain and genotyped with the S07_2966197 (A/G), S07_ 2970276 (G/C), and S07_ 2970381 (T/A) markers.

Haplotypes in SnAP	Alleles	Gene Pool Based on Q>85%	Resistant/Intermediate Accessions (No.)	Susceptible Accessions (no.)
**Haplo1**	AGT	Andean	−	73
		Andean admixed	−	22
		Middle American	−	2
		Middle American admixed	−	1
**Haplo2**	AGA	Andean admixed	3	–
		Andean	1	–
		Middle American admixed	1	–
**Haplo3-1**	ACA	Andean	68	–
		Andean admixed	55	–
		Middle American admixed	30	–
		Middle American	9	–
**Haplo3-2**	GCA	Middle American admixed	37	–
		Andean admixed	16	–
		Middle American	11	–
		Andean	7	–

**Figure 5:**
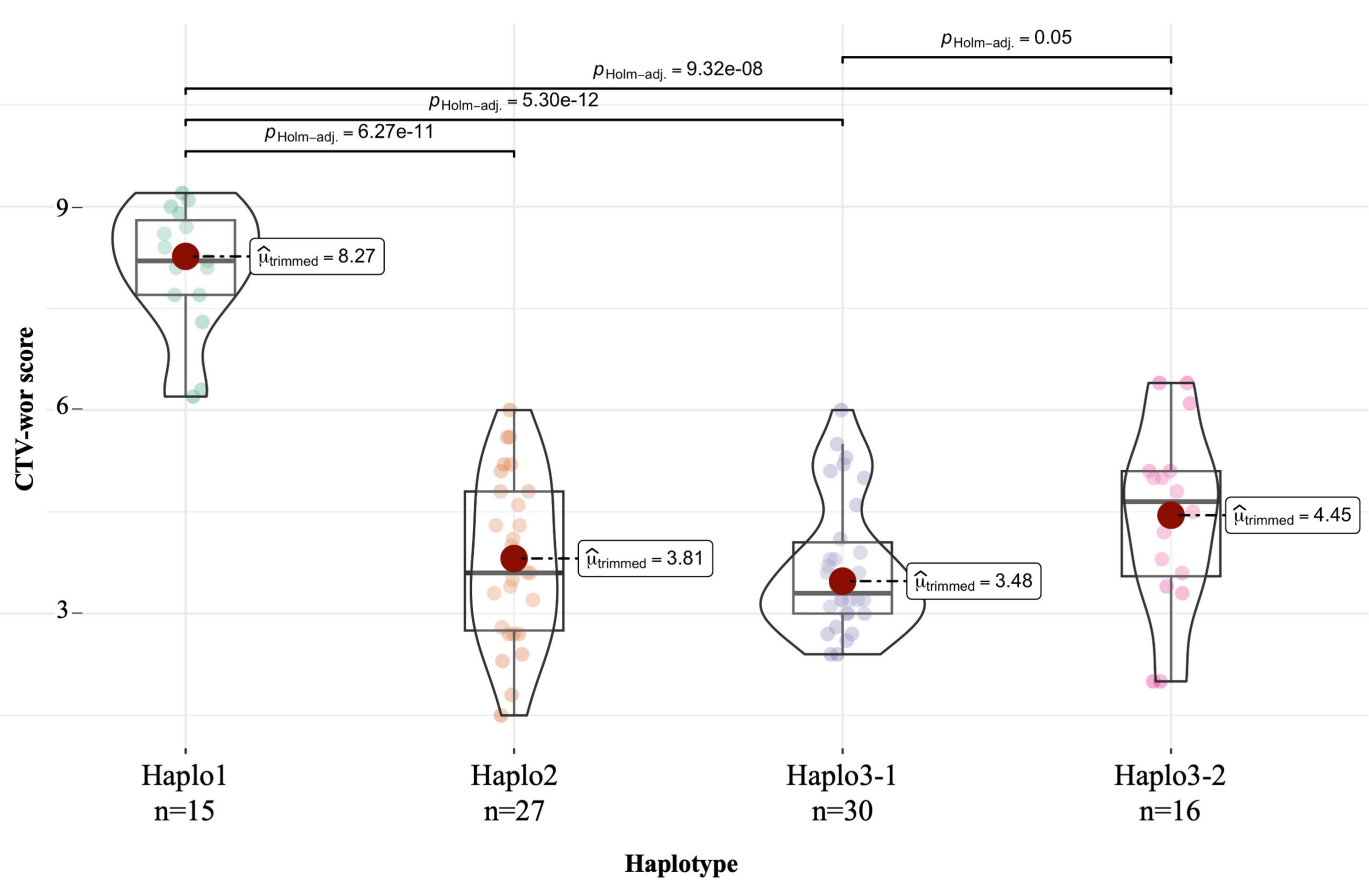
One-way ANOVA for quantitative CTV-Wor reaction (1 to 9 scale) in DBP accessions among four haplotypes compared by F_trimmed-means_ using the ggbetweenstats function in the R-Package ggstatsplot. Haplotypes based in polymorphism combination of S07_2966197, S07_2970276, and S07_297038 SNPs markers.

For the DBP (**Table 5**), Haplo1 was found in 15 Andean accessions with a mean disease score of 8.0, 13 with susceptible reactions and 2 tolerant accessions, ‘SVS-0683’ and ‘Beluga’ with scores of 6.3 and 6.2, respectively. Haplo2 was observed in 27 resistant/tolerant Andean accessions (3.8 mean score). There were 30 resistant/tolerant accessions (3.5 mean score) with Haplo3-1, 63% Middle American and 37% Andean. For Haplo3-2, 16 resistant/tolerant accessions (4.4 mean score), 62% Middle American and 38% Andean, were observed. CTV mean score comparisons among haplotypes in the DBP, using *p*_Holm_-adjusted *p*-values, showed that the susceptible Haplo1 was significantly different from the three resistant haplotypes (*p* < 0.0001), while the only significant difference among resistant haplotypes mean scores was between Haplo3-1, which was more resistant than Haplo3-2 (*p* = 0.05) (**Figure 5**).

**Table 5:** Haplotypes identified in DBP (88 accessions) phenotyped in the field with Worland strain and genotyped with the S07_2966197 (A/G), S07_ 2970276 (G/C), and S07_ 2970381 (T/A) markers.

Haplotype in DBP	Sequence	Gene Pool Based on Q>85%	Resistant/Tolerant Accessions (no.)	Susceptible Accessions (no.)	Trimmed Means
**Haplo1**	AGT	Andean	2	15	8.27
**Haplo2**	AGA	Andean	27	–	3.81
**Haplo3-1**	ACA	Middle American	19	–	3.48
		Andean	11	–	
**Haplo3-2**	GCA	Middle American	10	–	4.45
		Andean	6	–	

The haplotype survey using the same three markers was expanded to 57 dry bean genotypes, 30 Andean and 27 Middle American, which were agro-inoculated with the CA/Logan strain in Sun Prairie, WI (**Supplementary Table S10**). Both Haplo1 and Haplo2 were composed of only Andean genotypes, 7 with susceptible and 12 with resistant reactions to CTV, respectively. All Middle American genotypes were resistant to the CA/Logan strain and exhibited either Haplo3-1 (19 genotypes) or Haplo3-2 (8 genotypes). In addition, there were seven resistant Andean accessions with Haplo3-1 and 4 with Haplo3-2. Qualitative reactions to agro-inoculation with the CA/Logan strain matched the quantitative field reactions to Wor strain, for the 12 genotypes included in both the DBP and this additional survey, except for Beluga, which was susceptible to agro-inoculation but tolerant in the field.

## Discussion

The *Bct* gene region was first located to a 1.7-Mb interval on the proximal arm of chromosome Pv07 by GWAS using the GBS-generated SNP dataset for the SnAP panel agro-inoculated with the CA/Logan strain. A high LD block of the 50 most significant SNPs narrowed the *Bct* region to a 0.4-Mb interval. Six recombinant SnAP accessions assayed with designed SNPs further narrowed the interval to a 58-kb region. The ORF for five of the six gene models that spanned the 58-kb interval were sequenced across a select set of 28 snap and dry bean accessions. Of 33 SNP and InDel variants identified across the five genes, only one missense SNP (S07_2970381) in candidate gene Phvul.007G036300 (Exonuclease V) was identified as the most likely causal mutation conditioning *Bct* resistance. Moreover, GWAS of the DBP identified the S07_2970381 SNP variant as the only significant SNP for BCTV reaction in the field.

Exonuclease V has not been extensively studied in plants, and its function in viral resistance is yet to be elucidated. However, the *Arabidopsis* homolog AT5G60370, which encodes an Exonuclease V-Like (EXOVL) protein, exhibited 43% sequence identity (*E*-value = 1.76E−82) with the Phvul.007G036300 *Bct* candidate gene. EXOVL has been reported to play critical roles in biological processes underlying morphological traits in *A. thaliana* (Huang et al., 2022). Initially, Exonuclease V was identified and purified from *Saccharomyces cerevisiae*, encoded by the YBR163w gene (subsequently renamed EXO5). This protein is capable of degrading single-stranded DNA (ssDNA) from the 5′-end and plays a critical role in mitochondrial maintenance (Burgers et al., 2010). Furthermore, EXO5 is distantly related to the nuclease domain of RecB, a subunit of the bacterial RecBCD recombinase, which actively degrades linear double-stranded DNA (dsDNA), such as phage DNA, and thereby serves an antiviral function for the bacterium (Dillingham and Kowalczykowski, 2008).

The *Bct* EXO5 candidate gene is located 340.8 kb upstream from the BLC7.1 locus, which confers resistance to BLCrV, a Begomovirus (Ariza-Suarez et al., 2023). BCL7.1 is a minor-effect QTL that was associated with a Leucine-rich repeat receptor-like kinase (LRR-RLK) encoded by the gene model Phvul.007G040400 (Pv07: 3,312,090–3,315,269 bases). Ariza-Suarez et al. (2023) suggested previous breeding efforts for resistance to geminiviruses [e.g., CTV, bean golden yellow mosaic virus (BGYMV)] contributed to the accumulation of positive alleles against emerging viruses like BCL7.1. Although both *Bct* and BCL7.1 regions provide resistance to geminiviruses, a Curtovirus and Begomovirus, respectively, they do not physically overlap and exhibit low linkage disequilibrium. Perhaps, breeding for quantitative resistance to BGYMV, also a Begomovirus, conditioned by the BGY7.1 QTL spanning 2,724,611–3,525,083 bases on Pv07 (Soler-Garzón et al., 2021), contributed to the partial quantitative resistance observed against BLCrV. The broad genomic interval for BGY7.1 notably overlaps the *Bct* and BLC7.1 regions. Miklas et al. (2009) postulated that race Durango cultivars within the Middle American gene pool, such as ‘UI 35’ small red, ‘UI 114’ pinto, and others, which possess combined levels of resistance to CTV, BGYMV, and BDMV, could result from a common resistance gene or linked resistance genes located in the *Bct* region on Pv07. Continued research is warranted to further characterize and identify candidate genes within this “hot-spot” region, and mutations therein, that confer resistance to different geminiviruses.

The Tm-shift assay marker developed for SNP S07_2970381 will be useful for tracking CTV resistance conferred by the *Bct* EXO5 candidate gene in both snap and dry bean. The CTV reaction for 471 snap and dry bean accessions (excluding those segregating or missing data) from the SnAP, DBP, and additional survey of 57 dry bean genotypes clearly co-segregated with alleles of the S07_2970381 SNP marker. SVS-0863 dry bean with a tolerant disease score in the field but lacking the resistant marker allele is the only minor exception. The ‘Beluga’ dry bean with similar field tolerance but lacking the marker allele for resistance was later found to be susceptible to CTV *via* agro-inoculation.

The *Bct* EXO5 candidate gene is nearly ubiquitous in the Middle American gene pool as 98% (140/143) Middle American snap (including admixtures) and dry bean genotypes exhibited CTV resistance (including intermediate resistance and tolerance) and possessed the resistance allele for the EXO5 candidate gene mutation (S07_2970381 SNP). Conversely, only 65% (214/327) of Andean (including admixtures) snap and dry bean genotypes tested were resistant to CTV and possessed the resistance allele for the marker. In addition, all 113 Andean snap (including admixtures) and dry bean genotypes susceptible to CTV lacked the resistance allele for the *Bct* EXO5 candidate gene marker. Finally, only three Middle American snap bean accessions were susceptible to CTV, and they lacked the resistance allele for the marker.

The differences in percentage correlation between S07_2970381 and ‘SAS8_.1550_’, and S07_2970276 and ‘SAS8_.1550_’ markers, together with distinct haplotypes for combinations of the S07_2966197, S07_2970276, and S07_2970381 SNP markers in the SnAP and DBP, suggest different origins of the *Bct* candidate allele. The susceptible Andean beans all possess the same haplotype (Haplo1) for the three SNP markers S07_2966197, S07_2970276, and S07_2970381 in the *Bct* region. Haplo2 represents a small set of Andean genotypes with resistance to CTV. Among these genotypes, there are a few Andean dry bean landrace cultivars, including IJR (PI 163122, G8088) and G122 from India, Canadian Wonder (ADP_010) and Kijivu (ADP_033) from East Africa, Pompadour B from the Caribbean, and California LRK, with Haplo2, which suggests that this haplotype and associated *Bct* resistance is of Andean origin. This ‘Haplo2’ *Bct* resistance source was unlikely to have been purposely used in breeding for CTV resistance. All the dry bean genotypes in the DBP with Haplo2 represent mostly breeding lines that had never been screened for CTV reaction prior to this study, and their pedigrees reveal the use of Canadian Wonder, IJR, and Kijivu landrace cultivars as parents. Moreover, only 5 of 177 CTV-resistant snap bean accessions possessed Haplo2.Resistance to CTV in snap bean was purposely initiated from crosses with Middle American dry bean genotypes like ‘Burtner’ navy bean in 1936 and small red (~ ‘Common Red Mexican’) beans during the 1950s (Thomas and Zaumeyer, 1953). Haplo3-1 represents the source of *Bct* resistance from ‘Common Red’ landrace, and it is the most widely represented haplotype among resistant snap (70%) and dry bean (45%) genotypes in this study. Many of the dry bean genotypes with Haplo3-1 come from breeding programs that were actively selected for CTV resistance.

Haplo3-2, on the other hand, is represented by older great northern bean cultivars (i.e., UI-59, Harris, Beryl) and more modern race Durango cultivars (i.e., Matterhorn, Merlot, Kodiak) with upright architecture obtained from race Mesoamerica introgressions. Few, if any, of the dry bean genotypes with Haplo3-2 were developed by breeding programs with active or passive selection for *Bct* resistance. The mean disease score (3.5) for the Haplo3-1 dry bean genotypes indicates a significantly (*p* = 0.05) higher level of resistance than the mean score (4.4) for Haplo3-2 genotypes. Resistance levels for snap beans with Haplo2, Haplo3-1, or Haplo3-2 were undiscernible because only qualitative data existed for CTV reaction.

In light of these findings, breeding programs using S07_2970381 for MAS for CTV resistance conferred by the *Bct* EXO5 candidate gene should also include SNP markers S07_2966197 and S07_2970276 to monitor the different haplotypes and corresponding associations that they may have with quantitative levels of resistance observed by phenotyping. Meanwhile, based on information from this study, our recommendation is to use Haplo3-1 for MAS of CTV resistance.

## Data availability statement

The original contributions presented in the study are included in the article/**Supplementary Material**. Further inquiries can be directed to the corresponding author.

## Author contributions

AS-G, JH, and PM conceived and designed the experiments. AS-G and JH implemented statistic and bioinformatic analyses. DG, AT, and CS performed and conducted phenotyping strategies. QS performed SNP chip assay genotyping. KG conducted virus detection in the field. All authors contributed to the article and approved the submitted version.
