# Supplementary material for: A robust SNP-haplotype assay for Bct gene region conferring resistance to beet curly top virus in common bean (Phaseolus vulgaris L.)
Source: Front Plant Sci. 2023 Jul 14;14:1215950. doi: 10.3389/fpls.2023.1215950 (PMC10382175; doi:10.3389/fpls.2023.1215950)
Supplement: Supplementary file 2 [file DataSheet_1.pdf]

A B

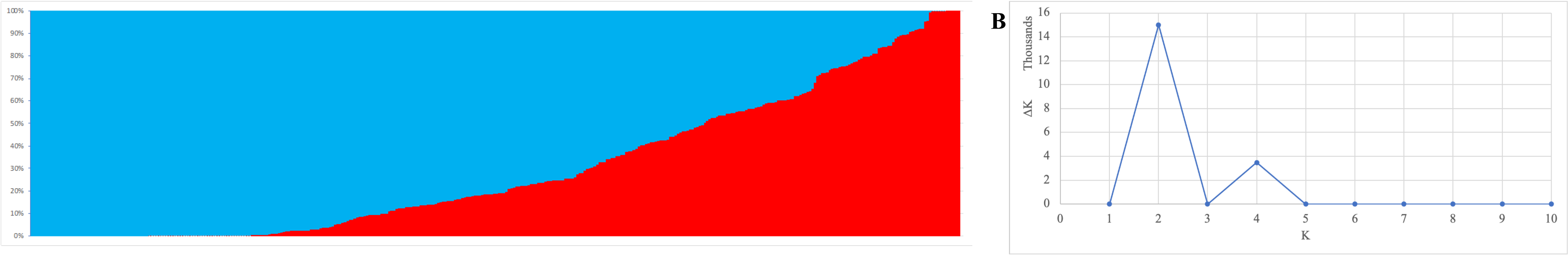

Figure S1. Model-based Bayesian clustering performed by STRUCTURE. **(A)** Each of the 378 SnAP accessions and two dry beans are separated by vertical bars into colored segments with lengths proportional to each of the *K* inferred ancestral populations. Blue color represents Andean genepool genepool and red color to Middle-American genepool, based on the membership coefficient (Q≥ 0.85). **(B)** Evano's delta K statistic determining an optimum of K=2 subpopulations.

A B

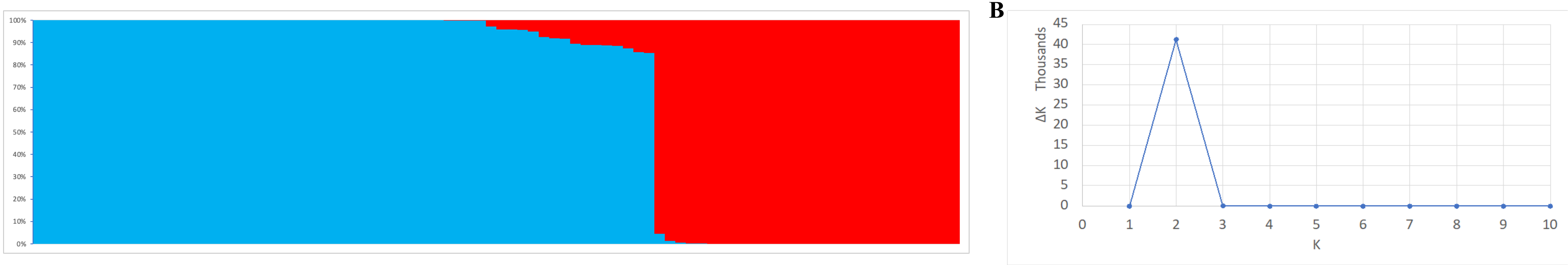

Figure S2. Model-based Bayesian clustering performed by STRUCTURE. **(A)** Each of the 88 dry beans genotypes are separated by vertical bars into colored segments with lengths proportional to each of the *K* inferred ancestral populations. Blue color represents Andean genepool genepool and red color to Middle-American genepool , based on the membership coefficient (Q≥ 0.85). **(B)** Evano's delta K statistic determining an optimum of K=2 subpopulations.

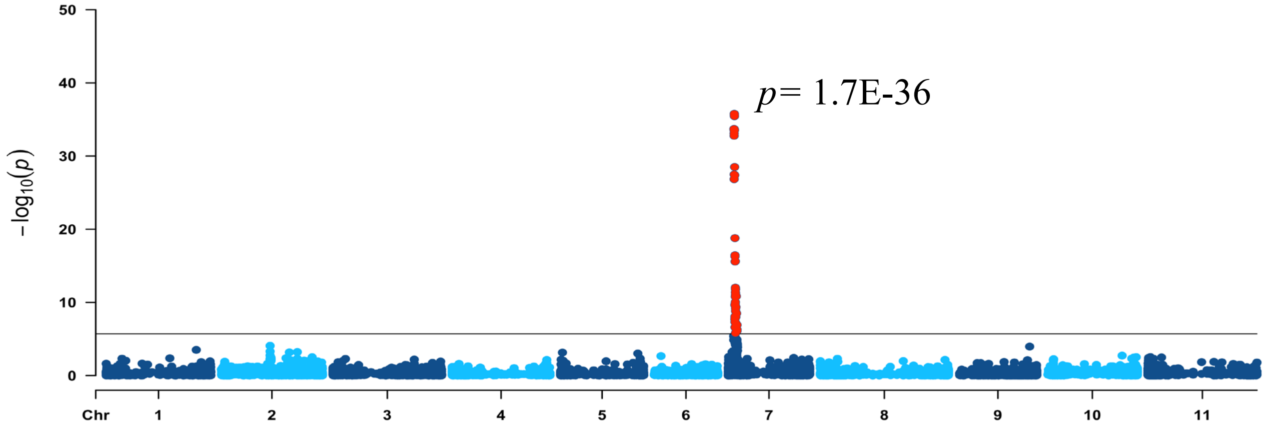

Figure S3a. Resistant (coded 1) vs. Intermediate Resistant (Coded: 2) vs. Susceptible (coded 0)

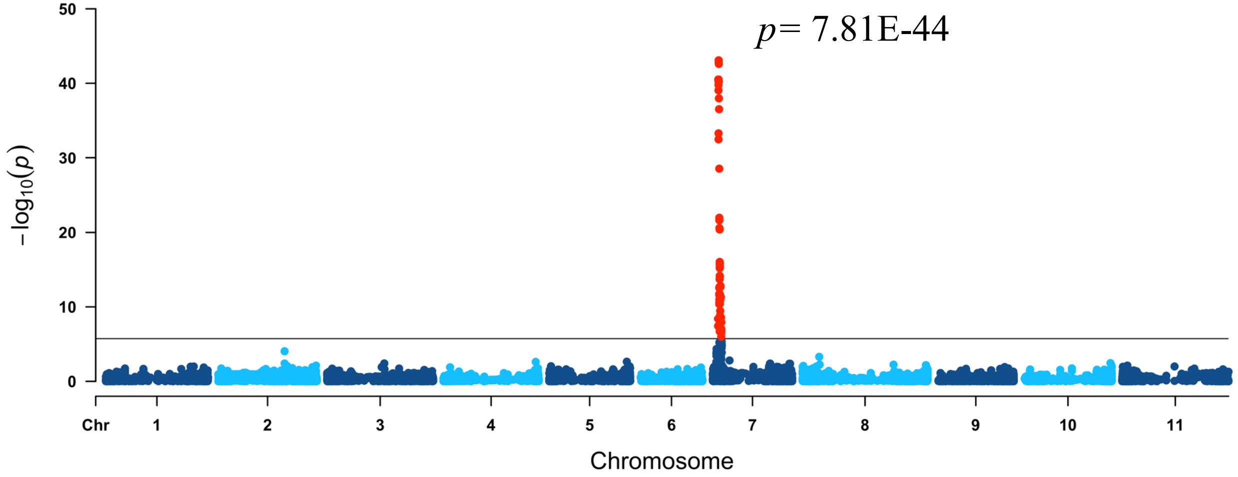

Figure S3b. Resistant (coded 1) vs. susceptible (coded 0) SnAP accessions

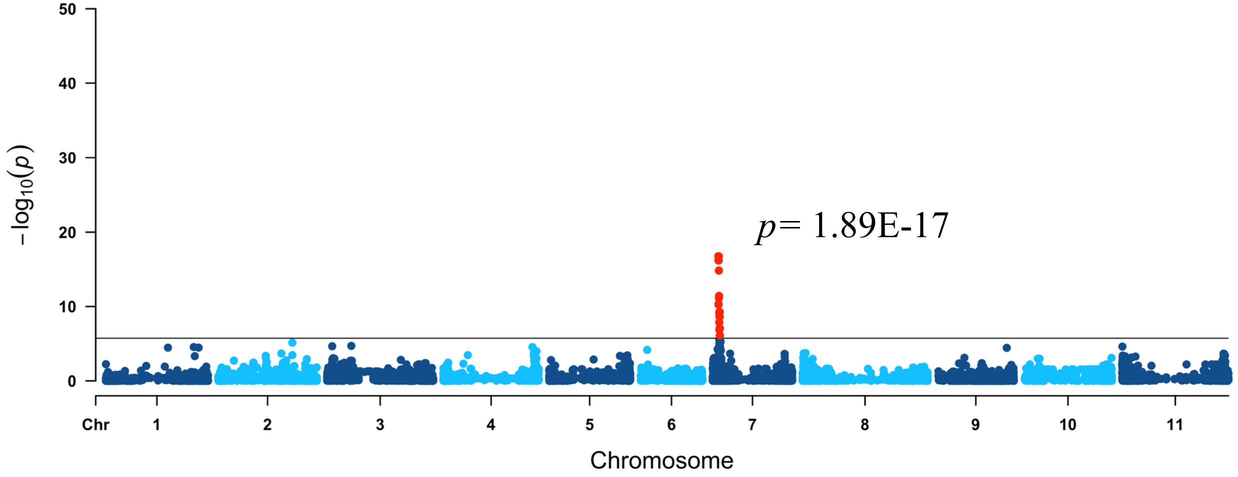

Figure S3c. Intermediate Resistant (coded 2) vs. susceptible (coded 0) SnAP accessions

A. S07_2966197
Ta = 68 ºC

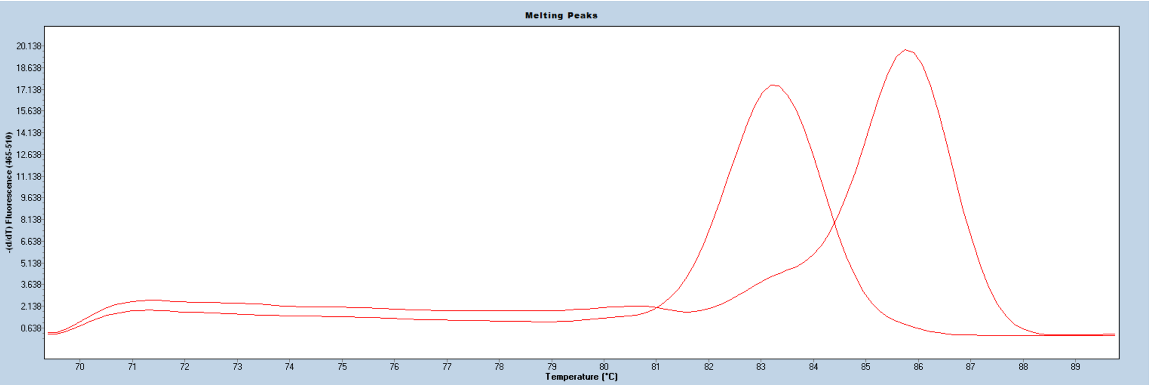

Othello (R) = 82
Matterhorn (T) = 84

B. S07_2970276
Ta = 58 ºC

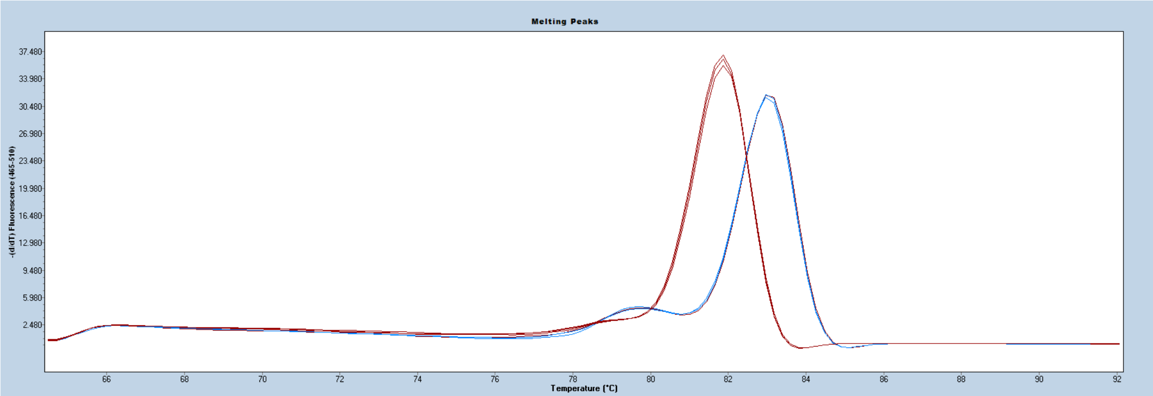

BAT 93 (R) = 82
Jalo EEP 558 (S) = 83

C. S07_2970381
Ta = 58 ºC

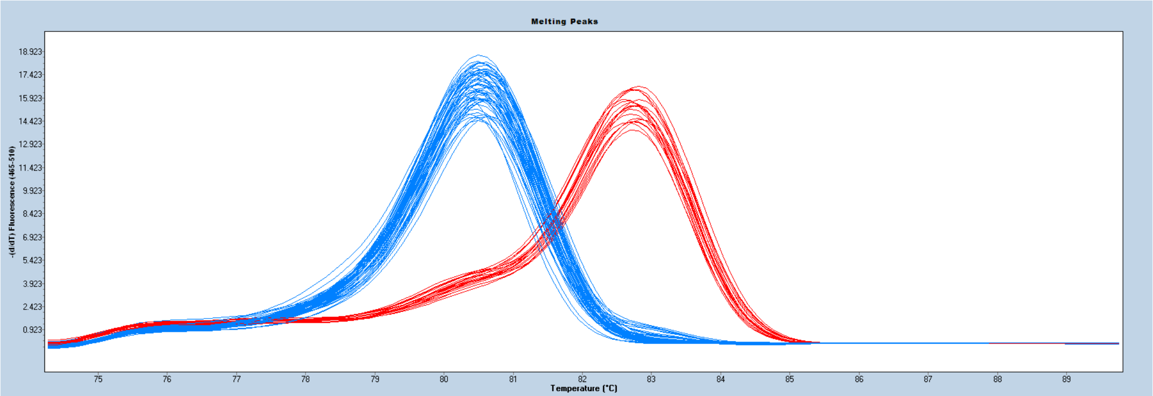

BAT 93 (R) = 80.5
Jalo EEP558 (S) = 83

Figure S4. Melting curve analysis. The melting curve of the three representative SNPs identified in *Bct* candidate genes that were genotyped with the Tm-shift method is shown here. A. S07_2966197; B. S07_2970276; C. S07_2970381.
